# Food intake and gestational weight gain in Swedish women

**DOI:** 10.1186/s40064-016-2015-x

**Published:** 2016-03-29

**Authors:** Linnea Bärebring, Petra Brembeck, Marie Löf, Hilde K. Brekke, Anna Winkvist, Hanna Augustin

**Affiliations:** Department of Internal Medicine and Clinical Nutrition, Sahlgrenska Academy, University of Gothenburg, Box 459, 405 30 Gothenburg, Sweden; Department of Biosciences and Nutrition, Karolinska Institutet, NOVUM, 141 83 Huddinge, Sweden

**Keywords:** Gestational weight gain, Dietary intake, Food groups

## Abstract

**Background:**

The objective of this study was to investigate if food intake (dairy, snacks, caloric beverages, bread, cheese, margarine/butter, potato/rice/pasta/grains, red meat, fish and fruit/berries/vegetables) is associated with gestational weight gain (GWG) in Swedish women.

**Methods:**

Four day food records from 95 pregnant Swedish women were collected in the last trimester. GWG was calculated as weighed body weight in the last trimester (median gestational week 36) minus self-reported pre-pregnancy body weight. Excessive GWG was defined according to the guidelines by the Institute of Medicine. Food groups tested for association with GWG were dairy (milk, yoghurt and sour milk), snacks (sweets, crisps, popcorn, ice cream and cookies, but not nuts and seeds), caloric beverages (soft drinks, juice, lemonade and non-alcoholic beer), bread, cheese, margarine/butter, potato/rice/pasta/grains, red meat, fish and fruit/berries/vegetables.

**Results:**

Median (lower–upper quartiles) GWG was 12.1 kg (10.0–15.3). In total, 28 % had an excessive GWG. Excessive GWG was most common among pre-pregnancy overweight and obese women, where 69 % had an excessive GWG. Median daily intake of fruits and vegetables was 352 g (212–453), caloric beverages was 238 g (100–420) and snacks was 111 g (69–115). Multivariable linear regression analysis showed that intake of caloric beverages, snacks, fish, bread and dairy in the last trimester of pregnancy were positively related to GWG (R^2^ = 0.32). Multivariable logistic regression analysis showed that intake of caloric beverages, snacks, fish, and bread was associated with higher odds ratios for excessive GWG.

**Conclusion:**

Intake of caloric beverages, snacks, fish and bread were positively related to excessive GWG. Thus, these results indicate that maternal dietary intake should be given higher attention in the antenatal care.

## Background

Excessive gestational weight gain (GWG) is associated with adverse maternal and fetal health outcomes. Women who gain more weight during pregnancy than recommended by the Institute of Medicine (IOM 2009) according to their pre-pregnancy body mass index (BMI) face higher incidences of gestational diabetes, pregnancy-induced hypertension and pre-eclampsia (Villamor and Cnattingius 2006), and their children face higher incidences of macrosomia and still birth (Villamor and Cnattingius 2006), as compared to mothers who gain within or less than recommended GWG (Rasmussen and Yaktine 2009).

In the last decade, average GWG has increased and excessive GWG (GWG above the IOM guidelines) is more common, especially among women with pre-pregnancy overweight (IOM 2009). Previous research show that excessive GWG among overweight women can be predicted as early as in the second trimester of pregnancy (Chmitorz et al. 2012). Excessive GWG is also associated with higher post-partum weight retention (Haugen et al. 2014), thus increasing the risk of entering a consecutive pregnancy with a higher BMI. In Sweden, early pregnancy BMI has been increasing since 1992 when weight and height started to be recorded in the medical charts of the antenatal care. In 2012, 25 % of women were overweight and 12 % obese at registration at their antenatal care clinic (The National Board of Health and Welfare 2013).

Due to the risks accompanied by excessive GWG, it is an important challenge to limit the trend of its increasing incidence. Determining factors of GWG are complex and preventive actions need to target modifiable lifestyle factors, such as dietary intake and physical activity (Stuebe et al. 2009). A meta-analysis of 12 intervention studies showed that change in diet and physical activity was effective in reducing GWG, but that the heterogeneity in outcomes was considerable (Gardner et al. 2011). A systematic review of observational studies showed that GWG was positively associated with intake of energy, protein and animal lipids, as well as energy density and number of food servings per day (Streuling et al. 2011). Intakes of carbohydrates and vegetarian diet were associated with lower GWG in the same study. Observational studies that link GWG to intake of certain foods are scarce. Previous studies have found that eating sweets and fast food (Olafsdottir et al. 2006; Shin et al. 2014; Uusitalo et al. 2009) and drinking milk increase risk of excessive GWG (Olafsdottir et al. 2006; Stuebe et al. 2009). However, these studies have mainly focused on dietary patterns or healthy eating indexes by scoring food frequency questionnaires, making it difficult to discern individual foods that are associated with GWG.

We hypothesize that higher intake of certain foods or food groups prevent or promote GWG by influencing the total energy intake. The objective of this study was to investigate if food intake is associated with GWG in Swedish women.

## Methods

### Participants

In total, 95 pregnant women were recruited from July 2008 to July 2011, to participate in a prospective observational study (Brembeck et al. 2013). Recruitment was carried out through posters at antenatal care clinics and other public places in Gothenburg, Sweden, or at a web page directed towards pregnant women. Inclusion criteria were that the participants should perceive themselves as healthy, having an uncomplicated pregnancy, in gestational week 35–37 at the study visit, between 25 and 40 years of age and not taking any prescription drugs that are known to affect bone metabolism. The study was planned and performed according to the guidelines in the Declaration of Helsinki and all procedures are approved by the Regional Ethics Committee in Gothenburg (Dnr 129-08). Written informed consent was obtained from all participants. The study was carried out at the Department of Internal Medicine and Clinical Nutrition at the University of Gothenburg, where all measures were conducted.

### Anthropometry

In gestational week 35–37, at the study visit, measurement of body weight after an overnight fast were performed using an electronic scale (Tanita, BWB-800MA; Rex Frederiksbergs Vaegtfabrik) and measurement of height performed using a standardized wall stadiometer. Body weight before pregnancy was self-reported at recruitment (the majority in first trimester and only few later in pregnancy). Gestational weight gain was calculated as weighed body weight in the third trimester minus self-reported pre-pregnancy body weight. Pre-pregnancy BMI was calculated using self-reported pre-pregnancy body weight and measured height. Excessive GWG was based on the guidelines by the IOM, based on pre-pregnancy BMI (IOM 2009).

### Food intake

At the study visit participants were instructed to complete a 4-day food record (four consecutive days, including at least 1 weekend day) starting as soon as possible after the study visit in the third trimester. Each participant received thorough instructions to accurately describe and quantify each meal, drink and food item consumed by either weighing the food, measuring the volume of food using household measures, or by using photographs of portion sizes of different foods and dishes (The National Food Agency 1997). Participants were also encouraged to include recipes when they consumed a mixed meal containing several components, such as soup or casserole, or food labels if the mixed meals were bought readymade. The participants were also encouraged not to change their eating behavior during the 4 days of dietary recording. Both verbal and written instructions were given by trained study personnel at the study visit. As soon as the food record was handed in (generally within a couple of weeks), it was checked to find any unclear information on type or amount of foods consumed. In such cases, the participants were contacted for additional information. Daily intake of food classified into groups was calculated using computer software Dietist XP version 3.1 (Kost och näringsdata, Bromma, Sweden).

Foods consumed were classified into ten groups in line with Swedish national food intake data (Amcoff et al. 2012): (1) dairy (milk, yoghurt and sour milk), (2) snacks (sweets, cakes, biscuits, potato chips, popcorn, but excluding nuts and seeds), (3) caloric beverages (soda, lemonade, juice and non-alcoholic beer, excluding milk), (4) bread, (5) cheese (including soft spread cheese and dessert cheese), (6) margarine and butter, (7) potato/rice/pasta/grains (not French fries or gratins), (8) red meat, (9) fish and (10) fruit/berries/vegetables. Dishes without recipes or approximate content were excluded.

### Physical activity level and energy expenditure

A ten-graded scale was used to estimate PAL, where one on the scale corresponded to PAL 1.3 and ten to PAL 2.2. The scale has been validated versus PAL assessed by the doubly labelled water method in combination with indirect calorimetry in non-pregnant women of childbearing age (Bexelius et al. 2010). Also in pregnant women, a moderate correlation has been found between estimated PAL using the 10-graded scale and PAL using the criterion method (r = 0.55, p = 0.008, unpublished results by M Löf). Basal metabolic rate (BMR) was calculated using Henry’s formula for women (Henry 2005), based on pre-pregnancy body weight. Here, 20 % was added to the BMR to account for increments related to pregnancy (Melzer et al. 2009).

### Statistics

Medians and lower and upper quartiles are presented when variables were not normally distributed. Simple and multivariable linear regression analyses were performed to test the association between the dependent variable GWG, and the food groups (grams per day of dairy, snacks, caloric beverages, bread, cheese, margarine/butter, red meat, fish, potato/rice/pasta/grains and fruit/berry/vegetables). The same independent variables were entered into a logistical regression analysis with GWG as a dichotomous dependent variable (non-excessive GWG = 0 and excessive GWG = 1). Age, parity, gestational week, PAL and pre-pregnancy BMI were considered potential confounders. A variable was considered a confounder if its inclusion in the model caused a beta coefficient to change >10 %. Food intake level (FIL: energy intake/BMR) was also considered a confounder to adjust for potential underreporting of food intake. Statistical analyses were carried out using SPSS version 22.0.0. Statistical significance was accepted at p ≤ 0.05. There was no missing data in variables used in the dataset.

## Results

Participant characteristics are shown in Table 1. Median age of the pregnant participants was 32 years and the study visit took place in median gestational week 36. Median pre-pregnancy BMI was 22 kg/m^2^, and BMI in the last trimester 26.4 kg/m^2^. Of the women, 93 % had studied at university level and 80 % had studied at university level for 3 years or more.

**Table 1:** Characteristics of the 95 pregnant Swedish women

	Median	Quartiles		Min	Max
		Lower	Upper		
Age (years)^a^	32.1	30.8	35.3	25.5	40.1
Parity	0	0	1	0	2
Physical activity level^b^	1.6	1.5	1.7	1.3	2.2
Gestational week at visit	35.9	35.1	36.4	32.1	37.9
Height (cm)^a^	168.5	164.8	173.0	154.0	184.0
Weight (kg)^a^	75.8	71.0	84.6	58.0	105.0
BMI (kg/m^2^)^a^	26.4	24.9	29.2	21.6	36.5
Pre-pregnancy weight (kg)^c^	63.0	59.0	69.0	50.0	94.0
Gestational weight gain (kg)^d^	12.1	10.0	15.3	3.7	29.3
BMI pre-pregnancy (kg/m^2^)^c^	22.2	20.7	23.7	18.0	31.3

^a^In the third trimester of pregnancy

^b^In the third trimester of pregnancy, estimated on a 10 graded scale

^c^Pre-pregnancy weight is self-reported

^d^Weight at study visit minus self-reported pre-pregnancy weight

### Gestational weight gain

In general, median GWG was 12.1 kg at the study visit in median gestational week 36, and 28 % (n = 27) had gained above the IOM recommendations for total GWG (IOM 2009). Most women (84 %, n = 80) had a normal BMI before pregnancy while 12 % (n = 11) were overweight, 2 % (n = 2) obese and 2 % (n = 2) were underweight. Median GWG among women with normal pre-pregnancy BMI was 12.1 kg, and median GWG for overweight women was 14.7 kg. Median GWG for women with pre-pregnancy underweight and obesity was 11.4 and 6.9 kg, respectively. Among women with a normal pre-pregnancy BMI, 18 (23 %) had gained above the IOM recommendations for total GWG at their study visit. Among women with pre-pregnancy overweight and obesity 9 (69 %) had gained above the recommendation.

### Food intake

Median energy intake was 2404 kcal (Table 2) and median energy expenditure was 2580 kcal. The energy intake of the group corresponded to 93 % of total energy expenditure. Median FIL was 1.53 (1.35–1.67) and median PAL was 1.6 (1.5–1.7). Median food intake is shown in Tables 2 and 3.

**Table 2:** Dietary intake and energy expenditure of the 95 Swedish women in third trimester of pregnancy

	Median	Quartiles		Min	Max
		Lower	Upper		
Energy intake (kcal)	2404	2155	2676	1420	3445
BMR (kcal)^a^	1566	1519	1636	1414	1928
Total energy expenditure (kcal)^b^	2580	2384	2881	1869	3446
Food intake level^c^	1.53	1.36	1.7	0.9	2.1
Fiber intake (g)	24	19	29	9	49
Fat intake E%	35	31	37	24	48
Protein intake E%	15	14	17	10	19
Carbohydrate intake E%	50	47	54	39	65
Saturated fatty acid intake E%	14	12	15	8	20
Sucrose E%	9	7	12	3	18

^a^Basal metabolic rate (BMR) calculated based on pre-pregnancy weight × 1.2 to account for pregnancy (Henry 2005)

^b^Calculated as BMR times physical activity level (estimated on a 10 graded scale (Bexelius et al. 2010)

^c^Calculated as energy intake divided by BMR

**Table 3:** Intake of food groups for the 95 Swedish women in the third trimester of pregnancy

Food groups (g/day)	Median	Quartiles		Min	Max
		Lower	Upper		
Dairy^a^	335	232	515	0	780
Bread	99	63	129	0	243
Vegetables	136	89	183	0	373
Fruit and berries	208	114	299	0	871
Total fruit, berries and vegetables	352	220	453	70	1053
Red meat	38	15	66	0	153
Fish	31	0	56	0	160
Snacks^b^	111	69	155	9	314
Soft drinks and lemonade	75	0	245	0	850
Juice	100	25	225	0	625
Total caloric beverages^c^	238	100	420	0	1340

^a^Sour milk, yoghurt and milk

^b^Sweets, crisps, popcorn, ice cream and cookies

^c^Soft drinks, lemonade, juice and nonalcoholic beer

In the multivariable linear regression analysis (Table 4), caloric beverages (p = 0.003), snacks (p = 0.005), bread (p = 0.008), and dairy (p = 0.015) were positively related to GWG. Gestational week at weighing in last trimester, pre-pregnancy BMI and PAL were confounders and also included in the model. PAL was the only variable inversely associated with GWG (p = 0.006). When FIL was added as a confounder, fish intake was also positively associated with GWG. The fully adjusted model explained 32 % of the variation in GWG (adjusted R^2^ = 0.32). In the fully adjusted multivariable logistical regression analysis (Table 5), the same variables (caloric beverages, snacks, bread and fish), except for dairy products, were significantly related to higher OR for excessive GWG.

**Table 4:** Linear regression analysis of the foods associated with gestational weight gain in 95 Swedish women

	Univariable		Adjusted^a^		Adjusted^b^	
	B	P	B	P	B	P
Dairy (g/day)	0.004	0.151	0.006	0.015	0.008	0.004
Bread (g/day)	0.013	0.175	0.025	0.008	0.029	0.002
Fruit and vegetables (g/day)	−0.005	0.052	0.000	0.946	0.002	0.481
Fish (g/day)	0.003	0.839	0.022	0.098	0.028	0.036
Red meat (g/day)	0.010	0.407	0.004	0.688	0.009	0.396
Sweet and salty snacks (g/day)	0.024	0.004	0.023	0.005	0.029	0.001
Caloric beverages (g/day)	0.006	0.001	0.006	0.003	0.007	<0.001
Margarine and butter (g/day)	0.014	0.792	−0.089	0.081	−0.074	0.144
Cheese (g/day)	−0.002	0.871	−0.010	0.458	0.005	0.760
Rice, pasta, grains (g/day)	0.001	0.858	0.001	0.896	0.005	0.483
Physical activity level^c^	−7.296	0.003	−6.724	0.006	−7.366	0.002

^a^Adjusted for gestational week at weighing and pre-pregnancy body mass index (BMI)

^b^Adjusted for gestational week at weighing, pre-pregnancy BMI and food intake level (FIL). FIL was calculated as energy intake divided by basal metabolic rate (BMR). BMR was calculated based on pre-pregnancy weight × 1.2 to account for pregnancy (Henry 2005)

^c^In the third trimester of pregnancy, estimated on a 10 graded scale (Bexelius et al. 2010)

**Table 5:** Logistical regression analysis of the variables associated with excessive gestational weight gain in 95 Swedish women

	Univariable			Adjusted^a^			Adjusted^b^		
	OR	CI lower	CI higher	OR	CI lower	CI higher	OR	CI lower	CI higher
Dairy (g/day)	1.000	0.997	1.002	1.001	0.998	1.005	1.002	0.999	1.006
Bread (g/day)	1.006	0.997	1.016	1.012	0.999	1.024	1.016	1.002	1.031
Fruit, berries and vegetables (g/day)	0.998	0.995	1.001	1.000	0.996	1.004	1.001	0.997	1.006
Red meat (g/day)	0.995	0.983	1.007	0.992	0.977	1.007	0.995	0.980	1.011
Fish (g/day)	1.005	0.992	1.018	1.018	0.999	1.038	1.023	1.002	1.044
Snacks (g/day)	1.010	1.002	1.019	1.012	1.000	1.023	1.018	1.004	1.032
Caloric beverages (g/day)	1.002	1.000	1.004	1.002	1.000	1.005	1.004	1.001	1.007
Margarine and butter (g/day)	1.015	0.967	1.065	0.959	0.894	1.030	0.968	0.896	1.045
Cheese (g/day)	1.003	0.989	1.017	0.999	0.981	1.017	1.010	0.988	1.032
Rice, pasta, grains (g/day)	0.999	0.992	1.007	0.999	0.990	1.009	1.002	0.992	1.012
Physical activity level^c^	0.026	0.002	0.436	0.016	0.000	0.611	0.007	0.000	0.381

^a^Adjusted for gestational week at weighing and pre-pregnancy body mass index (BMI)

^b^Adjusted for gestational week at weighing, pre-pregnancy BMI and food intake level (FIL). FIL was calculated as energy intake divided by basal metabolic rate (BMR). BMR was calculated based on pre-pregnancy weight × 1.2 to account for pregnancy (Henry 2005)

^c^In the third trimester of pregnancy, estimated on a 10 graded scale (Bexelius et al. 2010)

## Discussion

The main finding in this study is that intake of caloric beverages, snacks, fish and bread are positively associated with excessive GWG in Swedish women. Our results are in line with previous findings from Finland, where a dietary pattern including (among other foods) white bread, sweets, snacks, juice and soft drinks was associated with higher GWG (Uusitalo et al. 2009). These foods could be considered “in between meal foods”. A systematic review (Streuling et al. 2011) found that the number of meals consumed per day during pregnancy positively related to GWG. The general recommendation by the Swedish National Food Agency is to increase food intake by “2 filling snacks and 1 portion of fruit” in the last trimester of pregnancy (The National Food Agency 2008). This recommendation might by higher than required for some women, especially those who have already gained considerable weight at earlier stages of pregnancy. Further research is needed to determine whether snacking plays a role in excess GWG or not. The findings in the current study relating dairy intake to GWG are in line with findings associating milk intake with GWG (Olafsdottir et al. 2006; Stuebe et al. 2009). Previous studies have also seen an association between GWG and consumption of foods with added sugar (Olafsdottir et al. 2006; Uusitalo et al. 2009), which is comparable to our results. The positive association found between intake of fish and both GWG and excessive GWG, has to our knowledge never been seen or investigated before. This finding may be due to that the objective of the original study was to investigate changes in bone mineral density post-partum, in relation to vitamin D status. It is possible that some women increase their intake of fish, due to increased awareness. It is also possible that women who had already gained too much weight were more likely to do so.

Excessive GWG already in gestational week 36 was apparent in 28 %, mainly in women who were overweight or obese prior to pregnancy. Among these women, 69 % had gained more than recommended according to IOM’s guidelines for GWG. It is likely that these women also continued to gain weight throughout the last weeks of their pregnancies. Excessive GWG was apparent in 23 % of pre-pregnancy normal weight women. This indicates that overweight and obese women are at higher risk of excessive GWG, which may increase the risk of complications during pregnancy and delivery. This is in line with previous findings of excessive GWG in different BMI categories according to IOMs guidelines (Holowko et al. 2015).

It is important to keep in mind that GWG in the current study was calculated based on self-reported pre-pregnancy weight. However, Henriksson and colleagues have shown that self-reported pre-pregnancy weight agrees well with weight in early pregnancy (Henriksson et al. 2014). The dietary data in the current study were provided by 4-day food records in the last trimester of pregnancy. Dietary intake has previously been shown not to change from the second to last trimester of pregnancy in Swedish women (Lof et al. 2009). Thus, the dietary intake in the third trimester is likely a reflection of the diet earlier in pregnancy as well.

Other limitations are that most women in the current study had a normal BMI and the proportion of women in the current study with pre-pregnancy overweight or obesity was smaller than among women of childbearing age in Sweden in general. The GWG for the women in the current study seems to be a slightly lower compared to nationwide registry data (Holowko et al. 2014), but this could be explained by the fact that body weight of women in the current study were weighed in gestational week 36 and not at term. Also, PAL in the current study was measured using a subjective method which could be considered a limitation. Excessive GWG in this study was defined in accordance with the IOM guidelines. These recommendations were created for the American pregnant population but Nordic data is included in the report, and the guidelines are likely to be relevant also in a Swedish population. The participants in the current study reported consuming more fruit and berries, bread, fish, dairy, juice and sugared beverages, but less red meat compared to the corresponding Swedish female population (31–44 years of age) in a large nationwide dietary survey (Amcoff et al. 2012). In addition, participants in the current study had higher education than the general population. Therefore, this sample of pregnant women may not be representative of the general population of pregnant women in Sweden.

As excessive GWG can be predicted already in the second trimester, early detection of women at risk is possible (Chmitorz et al. 2012). Our results suggest that limiting intake of caloric beverages, snacks, fish and bread could reduce the incidence of excessive GWG and is perhaps most relevant for women at risk of exceeding the guidelines. More detailed understanding of the associations between dietary intake and GWG is needed. A larger cohort study, preferably population based, could further clarify how dietary intake relates to GWG in general and to excessive GWG in particular.

In conclusion, intake of caloric beverages, snacks, fish and bread were positively related to excessive GWG. Thus, these results indicate that maternal dietary intake should be given higher attention in the antenatal care.
